# Enhanced differential expression statistics for data-independent acquisition proteomics

**DOI:** 10.1038/s41598-017-05949-y

**Published:** 2017-07-19

**Authors:** Tomi Suomi, Laura L. Elo

**Affiliations:** 1 Turku Centre for Biotechnology, University of Turku and Åbo Akademi University, FI-20520 Turku, Finland; 2 Department of Future Technologies, University of Turku, FI-20014 Turku, Finland

## Abstract

We describe a new reproducibility-optimization method ROPECA for statistical analysis of proteomics data with a specific focus on the emerging data-independent acquisition (DIA) mass spectrometry technology. ROPECA optimizes the reproducibility of statistical testing on peptide-level and aggregates the peptide-level changes to determine differential protein-level expression. Using a ‘gold standard’ spike-in data and a hybrid proteome benchmark data we show the competitive performance of ROPECA over conventional protein-based analysis as well as state-of-the-art peptide-based tools especially in DIA data with consistent peptide measurements. Furthermore, we also demonstrate the improved accuracy of our method in clinical studies using proteomics data from a longitudinal human twin study.

## Introduction

The onset of high-throughput technology has enabled us to quantify complex protein mixtures using mass spectrometers. A widely used option for obtaining a global protein profile for a sample is label-free *shotgun* proteomics where the mass spectrometer is operated in data-dependent acquisition (DDA) mode. In this technique, the most intense precursor ions from a survey scan are isolated and fragmented to produce tandem mass spectra (MS/MS or MS2), which are then matched against a database of known sequences for peptide identification. However, shotgun proteomics suffers from two major drawbacks; the frequent occurrence of undersampling and taking the MS/MS spectra outside of the elution peak. This leads to low reproducibility and, consequently, only a proportion of detectable peptides will be identified reliably^1^.

A complementary approach to shotgun proteomics is the targeted mass spectrometry, which utilizes selected reaction monitoring (SRM). This approach uses the capabilities of triple quadrupole mass spectrometers to filter and selectively monitor a specific molecular ion and their corresponding fragment ions generated by collisional dissociation. These precursor-fragment ion pairs, termed SRM transitions, are repeatedly measured and counted over time, enabling reproducible quantification of the target peptides^2, 3^.

An emerging technology, called data-independent acquisition (DIA), capitalizes on the strengths of both the shotgun and targeted methods by combining the reproducibility of SRM with the extensive number of proteins identified in shotgun proteomics^4–6^. DIA methods avoid the need of detecting individual precursor ions during the analysis since the MS/MS scans are collected systematically (*independently* without precursor information) throughout the acquisition process. Unfortunately, due to the lack of a clear association between the precursor and its fragments, the data processing becomes more challenging and requires tools specifically designed for this task.

So far, most of the efforts to analyse data from DIA experiments have focused primarily on pre-processing and protein quantification and less on statistical analysis^7–9^. For instance, mapDIA^10^ offers multiple filtering options for pre-processing the input data. While proper pre-processing and quality control are crucial steps in the analysis, another important step is the choice of the method for the downstream statistical analysis. Even though there are currently some tools available to specifically handle data obtained from DIA experiments, such as MSstats^11^ and mapDIA^10^, there is still room for improvements in their performance. Therefore, the goal of this study was to develop a tool that could reliably estimate differential protein expression between sample groups, especially in DIA studies.

**Figure 1:**
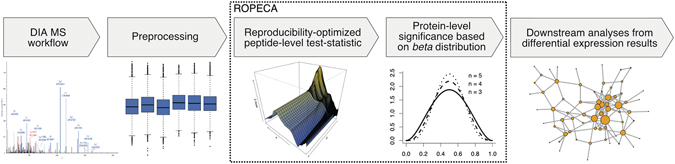
The general differential expression analysis workflow from experiment to downstream analysis. The dotted box highlights the ROPECA method, where the reproducibility-optimized test-statistic and the protein-level significance are calculated.

To this end we introduce here a new reproducibility-optimization method, called ROPECA (reproducibility-optimized peptide change averaging), which exploits all the peptide-level measurements when determining the protein-level changes. A schematic illustration of the workflow is shown in Fig. 1. ROPECA first optimizes the reproducibility of statistical testing for each data separately by maximizing the overlap of top-ranked features over group-preserving bootstrap datasets to enable robust analysis^12^ and then combines all available data from the peptide-level to improve the accuracy of the results by estimating the significance of median peptide-level *p*-values using the beta distribution^13^. This approach is different from tools, such as InfernoRDN (formerly known as DAnTE), which roll up already the peptide-level abundances before applying any statistical testing^14^. To demonstrate the benefits of ROPECA over the previously published approaches, we tested it with DIA data from two diverse benchmark studies as well as a clinical twin study. Although the benefits of our method are expected to be strongest when using DIA data with consistent peptide-level measurements, the method is also applicable to more conventional shotgun proteomics data, where the limitation, however, is the typically large number of missing values combined with small sample sizes.

## Results and Discussion

We first tested the applicability of our ROPECA method using the DIA profiling standard benchmark data, which contains 12 non-human proteins spiked into a constant human background in different concentrations^1^ (Table 1). These spike-in proteins are considered as true positives in our analysis. As expected, most background proteins had fold changes close to zero (Supplementary Fig. 1A).

**Table 1:** Summary of the relative concentrations of the spike-in samples in the DIA profiling standard used in assessing the performance of ROPECA.

	Master mix 1	Master mix 2	Master mix 3	Background
Sample 1	1	200	1	1
Sample 2	1.1	125.99	4	1
Sample 3	1.21	79.37	16	1
Sample 4	1.33	50	64	1
Sample 5	10	4	256	1
Sample 6	11.01	2.52	1024	1
Sample 7	12.11	1.59	4096	1

**Figure 2:**
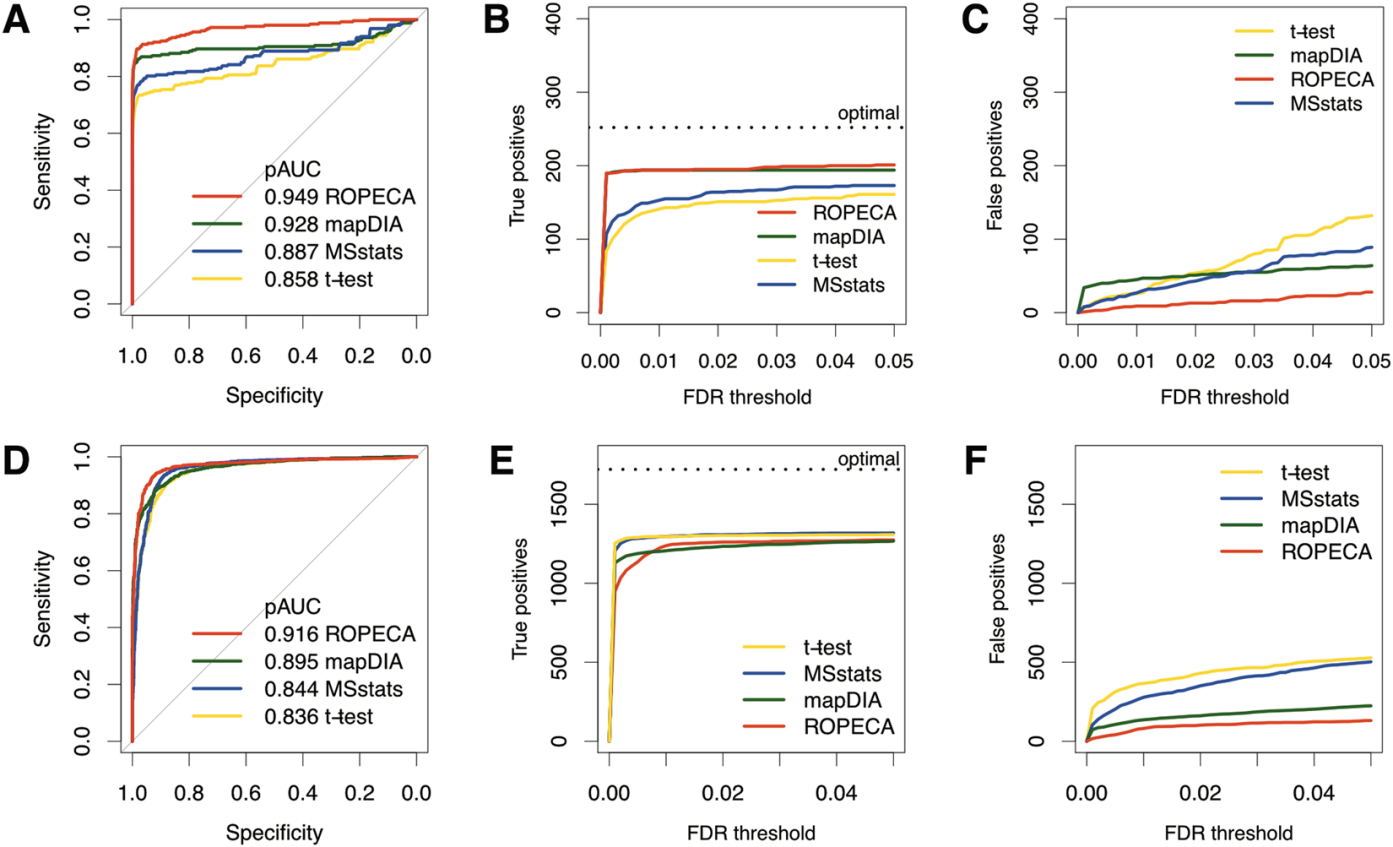
Performance of ROPECA in two diverse benchmark data sets. (**A)** Receiver operating characteristic (ROC) curves of the different statistical methods in the DIA profiling standard data. The ROC curves were produced by merging the individual results of all pairwise comparisons. The partial area under the curve (pAUC) for specificity above 0.9 is shown for each method. (**B**,**C)** Number of true positives and false positives in the DIA profiling standard as a function of false discovery rate (FDR) threshold. The black dotted line represents optimal performance. (**D)** ROC curves in the hybrid proteome benchmark data. (**E,F**) Number of true positives and false positives in the hybrid proteome benchmark data as a function of FDR threshold. The black dotted line represents optimal performance.

The performance of ROPECA, protein-level *t*-test, and peptide-based MSstats and mapDIA were investigated using receiver operating characteristic (ROC) curves (Fig. 2A), which were produced by merging the individual results of all pairwise comparisons from the seven sample groups tested (see Materials and Methods for details). To focus on the range of practical relevance, partial areas under the curves (pAUC) were calculated for specificity above 0.9 for all the methods. ROPECA produced significantly higher pAUC value of 0.949 than *t*-test (pAUC = 0.858), MSstats (pAUC = 0.887) or mapDIA (pAUC = 0.928), with bootstrap test *p* < 0.01 in all comparisons.

In addition to evaluating the ability of the methods to rank the proteins in order of evidence for differential expression, we also investigated the number of detected true positives and false positives as a function of false discovery rate (FDR) threshold from the same comparisons. This showed that ROPECA detected the largest number of true positives (Fig. 2B) while simultaneously reporting a low number of false positives (Fig. 2C), further demonstrating its effectiveness over the other methods. Comparison to our previously introduced probe-level method PECA^13^ and our protein-level reproducibility-optimization method ROTS^12^ showed the combined benefits of ROPECA with increased power to detect true positives like PECA while simultaneously reporting less false positives like ROTS (Supplementary Fig. 2). Investigation of the effect of the minimum intra-protein correlation filtering of the mapDIA software revealed that the filtering can both increase or decrease the performance of the methods depending on the selected parameters (Supplementary Fig. 3). However, none of the tested correlation filters on mapDIA resulted in higher pAUC values than the unfiltered ROPECA. This supports the robustness of our data-adaptive ROPECA and also demonstrates the difficulty in selecting the filtering parameters manually, when the underlying truth is not known, which is usually the case in real studies.

A data set with a large number of proteins that are up-regulated or down-regulated between the sample groups often reflects the actual biological changes more accurately than studies with just a small number of spike-in proteins like in the DIA profiling standard data. Therefore, we also tested ROPECA with a previously published hybrid proteome data^15^, where multiple organisms (human, yeast, and *E. coli*) were mixed together to create samples where 35% of the mixture contains proteins that are expected to be differentially expressed between the sample groups. These proteins are therefore considered as true positives in our analysis (Table 2). As expected, most of the other proteins had fold changes close to zero (Supplementary Fig. 1B).

**Table 2:** Summary of the sample composition in the hybrid proteome benchmark data.

	*E. coli*	Yeast	Human
Sample A	5%	30%	65%
Sample B	20%	15%	65%

Again, ROPECA produced significantly higher pAUC of 0.916 than *t*-test (pAUC = 0.836), MSstats (pAUC = 0.844) or mapDIA (pAUC = 0.895), with bootstrap test *p* < 0.01 in all comparisons (Fig. 2D). Although in terms of true positives, ROPECA performed slightly more conservatively than the other tested methods, at a typical FDR threshold of 0.05, the number of true positives reported by all the methods were roughly the same (Fig. 2E). In these data, the number of false positives was lowest with ROPECA, demonstrating its utility over the other methods (Fig. 2F). Similarly as in the DIA profiling standard data, ROPECA outperformed PECA and ROTS in terms of true and false positives (Supplementary Fig. 2).

**Figure 3:**
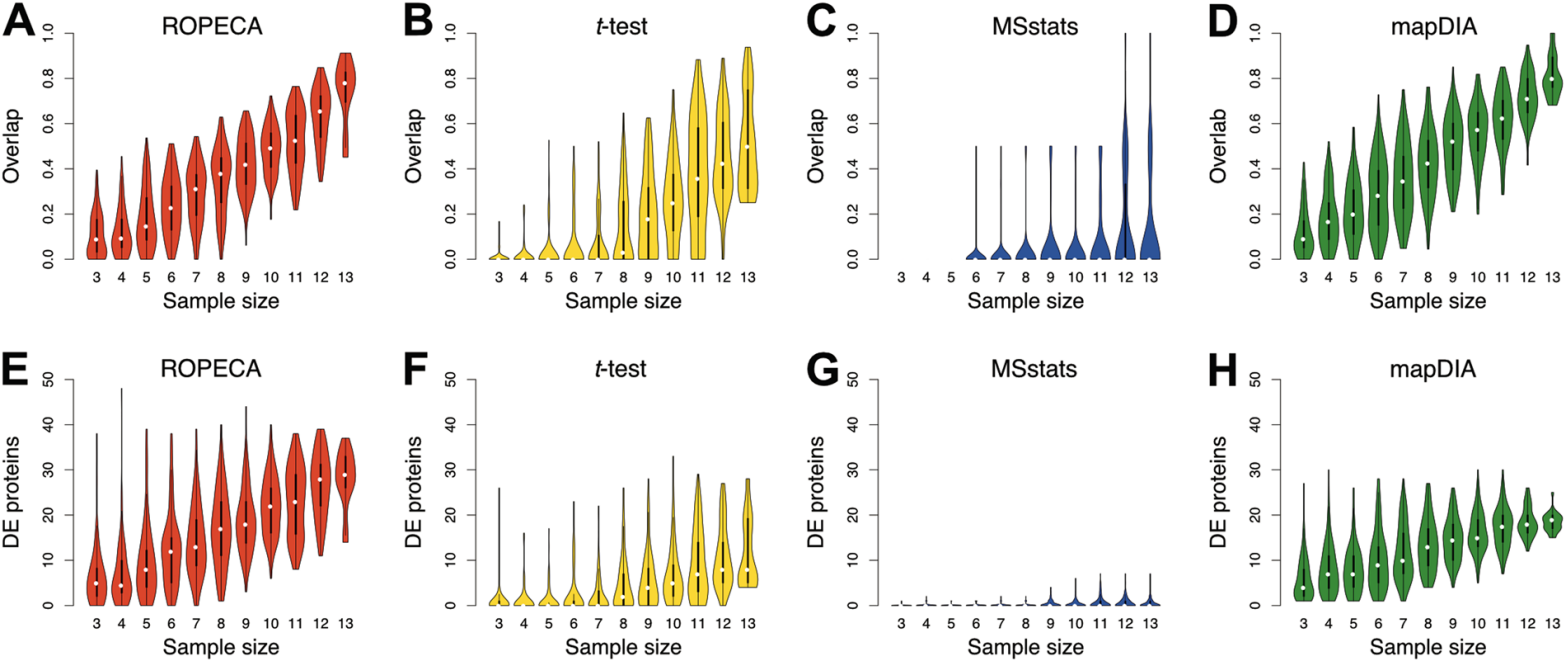
Reproducibility of the results in the clinical twin study. (**A–D)** Violin plots showing the proportion of overlapping differentially expressed proteins (FDR < 0.05) between the full data set (i.e. using all available samples) and 100 random subsets of 3–13 individuals diagnosed with type 2 diabetes (T2D) using ROPECA, *t*-test, MSstats, and mapDIA. Differential expression analysis was performed between subjects diagnosed with T2D and the rest of the study population (n = 102). (**E–H)** Violin plots showing the total number of differentially expressed proteins in the subsets analysed in Figure (**A–D**).

Finally, we wanted to test the utility of the ROPECA method on clinical data. For this we used a publicly available twin study data set, which was generated using the DIA technology^16^. Out of the 116 individuals, each having two follow-up visits, we separated those 14 individuals that were diagnosed with type 2 diabetes mellitus (T2D) from the rest of the population; differential protein expression was then tested between these two groups using measurements from the first visit. To test the reproducibility of the detections at FDR threshold of 0.05, we compared the results obtained using the full dataset against randomly sampled subsets, ranging in size from 3 to 13 (sampling without replacement). More specifically, we compared the proportion of common differentially expressed proteins identified in the full dataset with those found within a subset. Results of ROPECA (Fig. 3A), t-test (Fig. 3B), MSstats (Fig. 3C), and mapDIA (Fig. 3D) were illustrated using violin plots, each from 100 randomly sampled subsets, together with their total number of proteins reported within each subset (Fig. 3E–H). On average, ROPECA detected more proteins than *t*-test, MSstats and mapDIA (Wilcoxon signed rank test *p* < 0.05). It also retained a higher number of common detections than *t*-test or MSstats especially at smaller sample sizes (Wilcoxon signed rank test *p < *0.001 for all sample sizes when comparing ROPECA overlaps to *t*-test and MSstats overlaps). Overall, the highest relative overlap was produced by mapDIA. Notably, however, mapDIA produced significantly highest overlaps also in random mock comparisons generated by repeatedly splitting the data into two parts (Wilcoxon signed rank test *p < *0.001 over 100 random comparisons, Supplementary Fig. 4). This is likely due to mapDIA having a significant correlation between the estimated FDR and the number of peptides for each protein (Pearson’s product-moment correlation *p* < 0.001 over all the random comparisons).

To estimate the proportion of false positive detections in the twin study, we similarly tested differential expression between artificial sample groups, which were created by randomly splitting the data of the first visits into two parts. As none of the proteins were expected to be differentially expressed, it enabled us to estimate the accuracy of the FDR values reported by each method. For 100 randomly sampled runs using FDR threshold of 0.05, the estimated FDR from mock comparisons was 0.002 with ROPECA, 2 × 10^−5^ with both *t*-test and MSstats, and 0.004 with mapDIA, which suggests that all the estimates were conservative.

**Figure 4:**
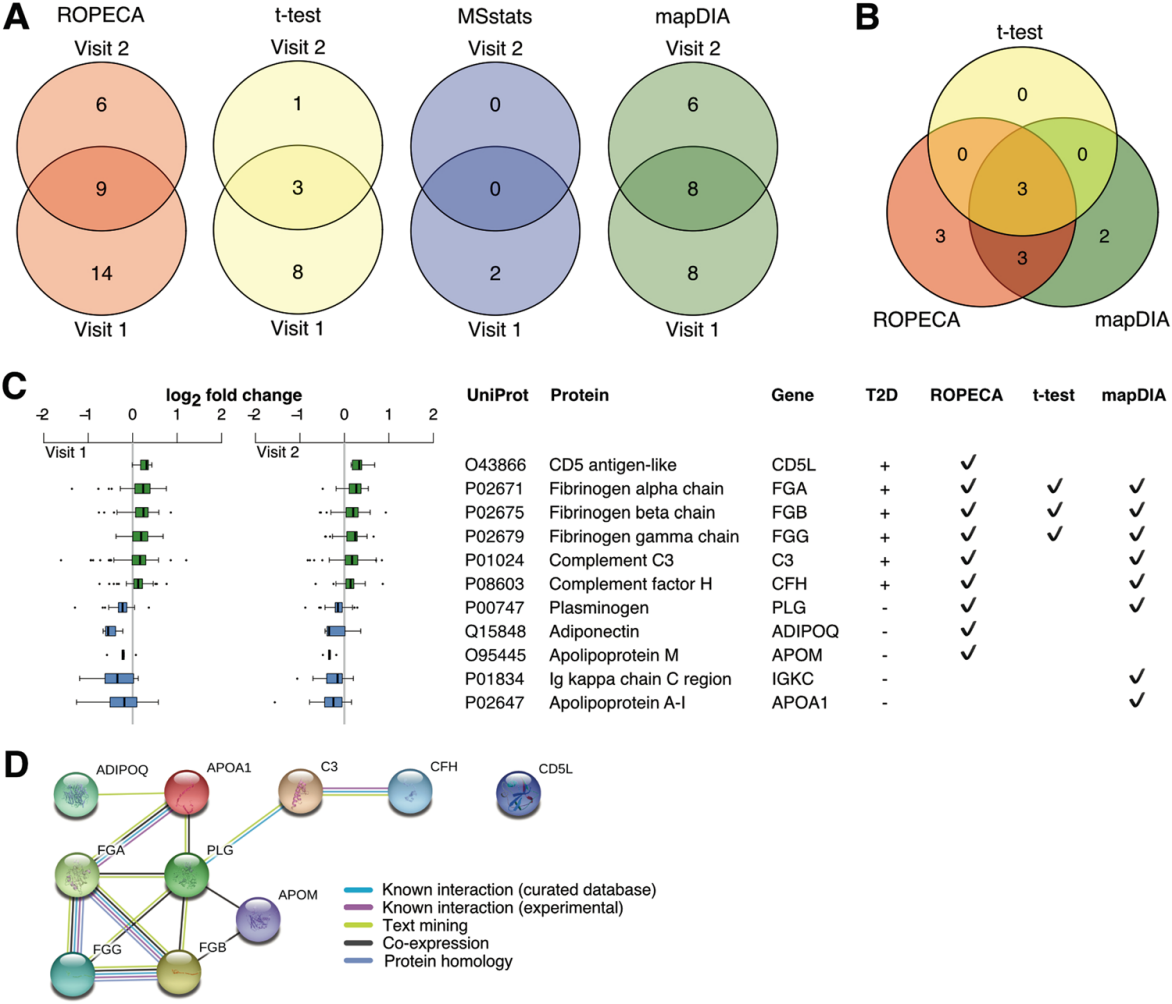
Differentially expressed proteins in type 2 diabetes (T2D). (**A**) Overlaps of differentially expressed proteins (FDR < 0.05) in the two follow-up visits using ROPECA, *t*-test, MSstats, and mapDIA. (**B**) Overlaps of the differentially expressed proteins between the different methods. MSstats was omitted as it did not report any proteins on both visits. (**C**) Fold changes of the differentially expressed proteins on both visits. Only proteins with fold change to the same direction on both visits and identified using more than a single peptide were included. (**D**) Known protein-protein interactions between the differentially expressed proteins shown in panel C reported in the STRING database.

Finally, we investigated the biomedical relevance of the differentially expressed proteins (FDR < 0.05) reported by each method between the 14 individuals diagnosed with T2D and the rest of the twin study population (n = 102) in both of the two follow-up visits. Ambiguous hits corresponding to multiple proteins or proteins detected using only a single peptide were filtered out. In addition, fold changes were required to be in the same direction on both visits. ROPECA identified a total of nine proteins as differentially expressed on both visits, *t*-test three, MSstats zero, and mapDIA eight proteins (Fig. 4A). Notably, the three proteins identified by *t*-test were also significant using both ROPECA and mapDIA, while three unique proteins were reported as significant by ROPECA and two by mapDIA (Fig. 4B).

Although the initial study was not aimed specifically to investigate T2D, the detected differentially expressed proteins (Fig. 4C) were well supported by existing literature. For example, increased plasma fibrinogen levels^17^ and increased levels of fibrinogen alpha, beta and gamma chains^18^ have been reported in T2D patients. Also, the plasma levels of C3 have been shown to be significantly associated with the development of diabetes^19^. Decreased levels of apolipoprotein M in plasma of diabetic patients has been reported in multiple studies^20, 21^; the same can be said for adiponectin^22, 23^. Moreover, 10 out of the 11 proteins were found in the STRING database^24^ and known protein-protein interactions were reported between nine of them (Fig. 4D). Finally, we confirmed that for none of the candidate proteins, the protein abundance was significantly correlated with subject age (*FDR* > 0.05; Supplementary Fig. 5).

To summarize, we have developed a new reproducibility-optimized peptide-based tool, ROPECA, for performing differential expression analysis on proteomics data. The method was tested and its benefits were illustrated using diverse DIA datasets. In the spike-in benchmark data and the hybrid proteome benchmark data, ROPECA systematically outperformed the currently accepted methods by reporting at least the same number of true positives as the other methods while simultaneously reporting less false positives at the typical FDR threshold of 0.05. Additionally, we showed the applicability of ROPECA in actual clinical data using a human twin study, where we identified several differentially expressed proteins supported by literature that would have been missed by conventional statistical testing. Besides enabling robust analysis of data that has been generated using various types of DIA approaches, the tool also works with conventional shotgun proteomics data. However, the performance is limited by the typically large number of missing values combined with a small sample size, making the bootstrap procedure of ROPECA less effective. There, our previously introduced PECA method is currently recommended (Supplementary Fig. 6). This approach, now shown to perform well using peptide-level measurements, could also be further extended to exploit the possible fragment-level measurements. ROPECA is implemented in the PECA R-package, which is freely available from Bioconductor (https://bioconductor.org/packages/PECA).

## Materials and Methods

### Analysis environment

For the analysis, the following tools were used: R 3.3.2, MSstats 3.6.0, PECA 1.10.0, ROTS 1.3.1, mapDIA 2.4.1, and genefilter 1.56.0. The scripts to perform the analyses are available from our website (http://www.btk.fi/research/research-groups/elo/downloads/).

### Reproducibility-optimized peptide change averaging (ROPECA)

ROPECA uses peptide-level statistics to infer differential protein expression following a similar concept as our previously introduced PECA method^13^. Unlike in PECA, however, the peptide-level test statistics are optimized to maximize the reproducibility of the detections. To achieve this, ROPECA uses the reproducibility-optimized test statistic (ROTS)^25^. More specifically, it optimizes the reproducibility by choosing a statistic from a family of *t*-type statistics that maximizes the overlap of top-ranked peptides in group-preserving bootstrap datasets. The modified *t*-statistic is calculated as:

1 $${d}_{\alpha }=\frac{|{\bar{x}}_{1}-{\bar{x}}_{2}|}{{\alpha }_{1}+{\alpha }_{2}s}\,$$

where $|{\bar{x}}_{1}-{\bar{x}}_{2}|$ is the difference between the two group averages of normalized peptide abundances, α_1_ and α_2_ are non-negative parameters to be optimized, and *s* is the pooled standard error. The optimal statistic is determined by maximizing the reproducibility *Z*-score:

2 $${Z}_{k}({d}_{\alpha })=\frac{{R}_{k}({d}_{\alpha })-{R}_{k}^{0}({d}_{\alpha })}{{s}_{k}({d}_{\alpha })}$$

over a lattice ${\alpha }_{1}\in \{0,\,0.01,\mathrm{...},5\}$, ${\alpha }_{2}\in \{0,1\}$, $k\in \{0,1,2,\mathrm{...},F\}$, where *F* is the total number of peptides in the data, ${R}_{k}({d}_{\alpha })$ is the reproducibility of statistic ${d}_{\alpha }$ at top list size *k* in bootstrap datasets, ${R}_{k}^{0}({d}_{\alpha })$ is the corresponding reproducibility in randomized datasets permuted over samples, and ${s}_{k}({d}_{\alpha })$ is the standard deviation of the bootstrap distribution. Reproducibility is defined as the average overlap of *k* top-ranked peptides over pairs of bootstrapped datasets.

For protein-level inference of differential expression, the median of peptide-level *p*-values is used as a score for each protein taking the direction of change into account. The protein-level significance of the detection is then calculated using beta distribution. Under the null hypothesis, the *p*-values of the peptides follow the uniform distribution *U*(0,1). Furthermore, the order statistics from *U*(0,1) distribution follow a beta distribution. More specifically, the *i* ^th^ order statistic of sample size *n* has a beta distribution *B*(*γ,δ*) with parameters *γ* = *i* and δ = *n* − *i* + 1 ^26^. The significance of the median *p*-value for a protein with *n* peptides is hence calculated using the cumulative distribution function of the beta distribution’s probability density function^27^:

3 $$\frac{1}{B(\gamma ,\delta )}{\int }_{0}^{{P}_{m}}{x}^{\gamma -1}{(1-x)}^{\delta -1}dx$$

where *P*_*m*_ is the observed median *p*-value of peptides belonging to the protein. Finally, the FDR is calculated using the Benjamini-Hochberg procedure.

### MSstats, mapDIA and *t*-test

Bioconductor package MSstats and SourceForge package mapDIA were applied to the normalized peptide-level data. In MSstats, the analysis was done using the default settings. No normalization was performed in the dataProcess function. In mapDIA, the experimental design was set as *IndependentDesign*. No normalization or imputation of data within the software was used. Standard deviation factor (SDF) was set to *inf* and median intra-protein correlation cutoff (MIN CORREL) was set to −1. Minimum number of observations for each group (MIN_OBS) was set to 1 and minimum number of peptides per protein (MIN_PEP_PER_PROT) was set to 1. These settings ensure that that no additional filtering was performed within the software and that the benchmark focuses only on the statistical model of the software. A two-sided two-group *t*-test with equal variances was applied using rowttests function in the Bioconductor genefilter package to the protein-level data obtained by summarizing the normalized peptide intensities. The Benjamini-Hochberg procedure was used to control the FDR of *t*-test. For MSstats and mapDIA, the FDR reported by the software was used.

### DIA profiling standard

For benchmarking purposes we used the DIA profiling standard of Bruderer *et al*.^1^, which contains 12 non-human proteins spiked into a constant human background (HEK-293) with different known concentrations in eight sample groups, each having three replicates. The DIA data used here were downloaded from the supplementary files of Bruderer *et al*. containing local regression normalized^28^ peptide quantifications preprocessed using Spectronaut^29^. For *t*-test the normalized peptide intensities were summed to protein-level. The DIA profiling standard is available from PeptideAtlas: No. PASS00589 (username PASS00589, password WF6554orn).

Results are shown after excluding sample group 8 from the analyses. There was large variability in the comparisons involving the sample group 8, and all the tested methods performed exceptionally poor in those comparisons. We hypothesized that a high abundance of some proteins in the sample (relative concentration of 16 384 times the background) had an impact on the quantitation of other proteins, thus leading to biased results. This effect is typical for DDA data^12^. By filtering out the group 8 from the analysis, we increased the performance of all the methods, especially reducing the number of false positives.

### Hybrid proteome data

We also used data from Kuharev *et al*.^15^ for benchmarking differential expression in DIA data. The data was originally used for testing three different software packages (ISOQuant, Progenesis and Synapter) for processing MS^E^ ^30^ and UDMS^E^ ^6^ DIA data. The data consists of two hybrid proteome samples of known composition, both with five technical replicates. The samples contain human, yeast and *E. coli* proteins, where only the proportion of yeast and *E. coli* proteins change between the two sample groups. This constitutes 35% of the total sample composition, thus reflecting an actual biological sample, where a large number of proteins can be differentially expressed. Raw and pre-processed data are available from the PRIDE Archive (id PXD001240). From all the available data, we used the UDMS^E^ based results, which were processed using ISOQuant software. For ROPECA, MSstats and mapDIA, the intensities of identical peptide sequences were first summed to form the peptide-level data and this peptide-level data was then median normalized. The same peptide-level data was used with each method to ensure comparability. For *t*-test, these median normalized peptide intensities were further summed to protein-level.

### Longitudinal clinical twin data

To demonstrate the performance of ROPECA in clinical proteomic studies, we used data from a longitudinal human twin study by Liu *et al*.^16^, which consists of human plasma proteins quantified using the SWATH technique^4^. The samples were collected longitudinally from two time points at intervals of 2–7 years from 72 monozygotic and 44 dizygotic twins. The preprocessed OpenSWATH data was downloaded from the PRIDE Archive (id PXD001064) and converted to suitable matrix format for statistical analysis using SWATH2stats R-package^31^. The peptide-level data was median normalized and used as input for ROPECA, MSstats and mapDIA analysis. For *t*-test, we used the available protein-level data, which was median normalized similarly.

## Electronic supplementary material

Supplementary
