# Supplementary material for: Enhanced differential expression statistics for data-independent acquisition proteomics
Source: Sci Rep. 2017 Jul 19;7:5869. doi: 10.1038/s41598-017-05949-y (PMC5517573; doi:10.1038/s41598-017-05949-y)
Supplement: Supplementary file 1 — Supplementary [file 41598_2017_5949_MOESM1_ESM.pdf]

Enhanced differential expression statistics for data-independent acquisition proteomics

Supplementary

Tomi Suomi[1,2,*], Laura L. Elo[1,*]

[1] Turku Centre for Biotechnology, University of Turku and Åbo Akademi University, FI-20520 Turku, Finland

[2] Department of Future Technologies, University of Turku, FI-20014 Turku, Finland

* Corresponding author: tomi.suomi@utu.fi, laura.elo@utu.fi

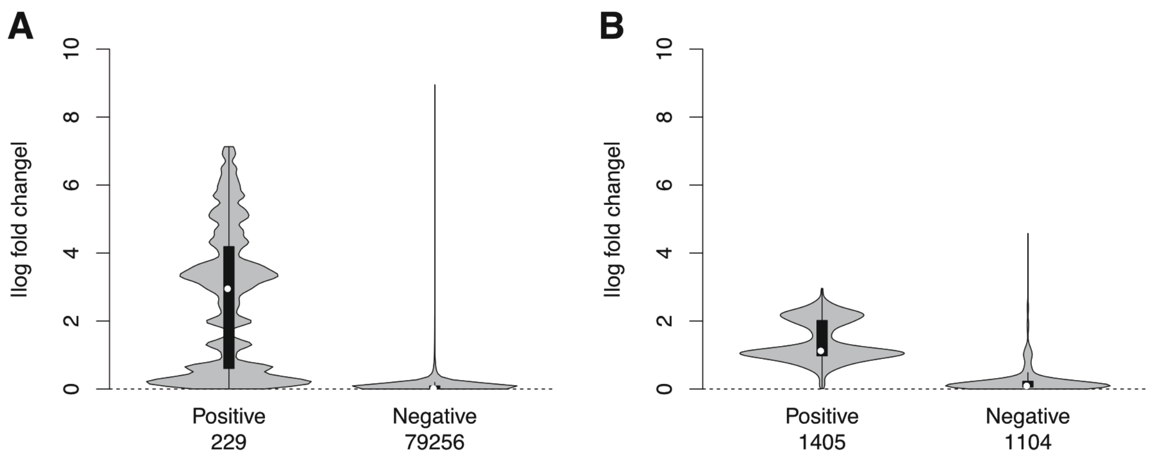

Supplementary Figure 1: Distributions of the fold change values for the known positives and the background negatives. **A**: Distributions in the DIA profiling standard data. **B**: Distributions in the hybrid proteome benchmark data.

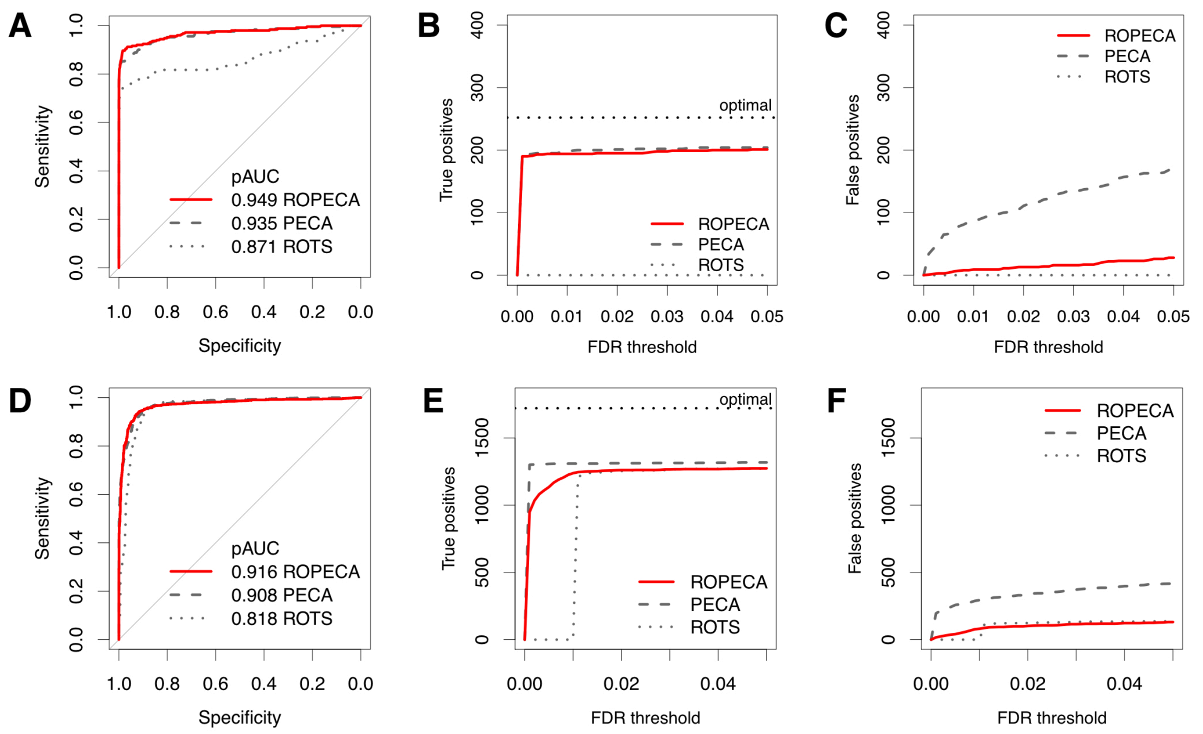

Supplementary Figure 2: Performance of ROPECA, PECA and ROTS in two diverse benchmark data sets. **A**: Receiver operating characteristic (ROC) curves of the different statistical methods in the DIA profiling standard data. The ROC curves were produced by merging the individual results of all pairwise comparisons. The partial area under the curve (pAUC) for specificity above 0.9 is shown for each method. **B, C**: Number of true positives (B) and false positives (C) in the DIA profiling standard as a function of false discovery rate (FDR) threshold. The dotted black line represents optimal performance. **D**: ROC curves in the hybrid proteome benchmark data. **E, F**: Number of true positives (E) and false positives (F) in the hybrid proteome benchmark data as a function of FDR threshold. The dotted black line represents optimal performance.

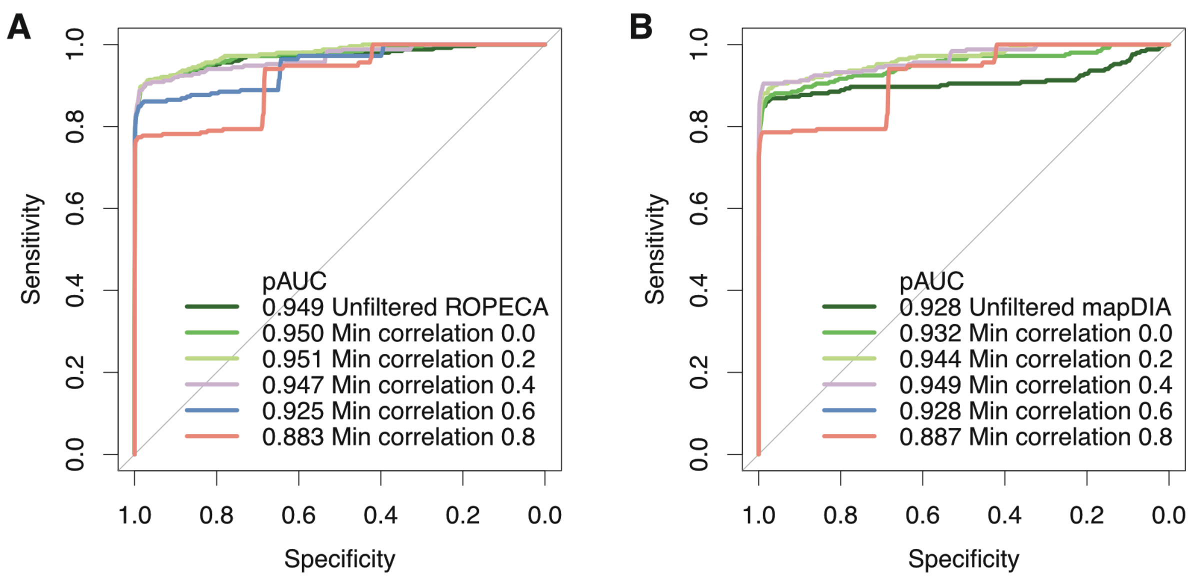

Supplementary Figure 3: Performance in the DIA profiling standard data when applying different criteria for the minimum correlation filtering implemented in the mapDIA software. Receiver operating characteristic (ROC) curves were produced by merging the individual results of all pairwise comparisons. The partial area under the curve (pAUC) for specificity above 0.9 is shown for the unfiltered data and the different filtering criteria. **A**: Performance of ROPECA. **B**: Performance of mapDIA.

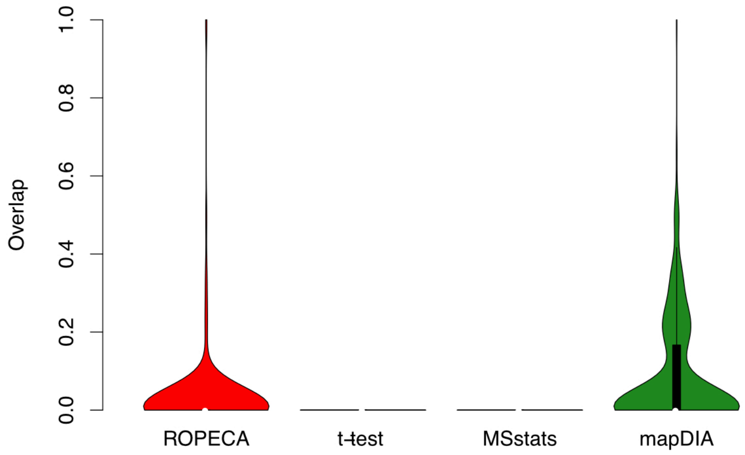

Supplementary Figure 4: Reproducibility of the results in the clinical twin study data after randomization of the sample groups. The violin plots show the proportion of overlapping differentially expressed proteins (FDR < 0.05) between 100 pairs of datasets, each generated by randomly splitting the data of the first visits into two parts. None of the proteins were expected to be differentially expressed in these comparisons.

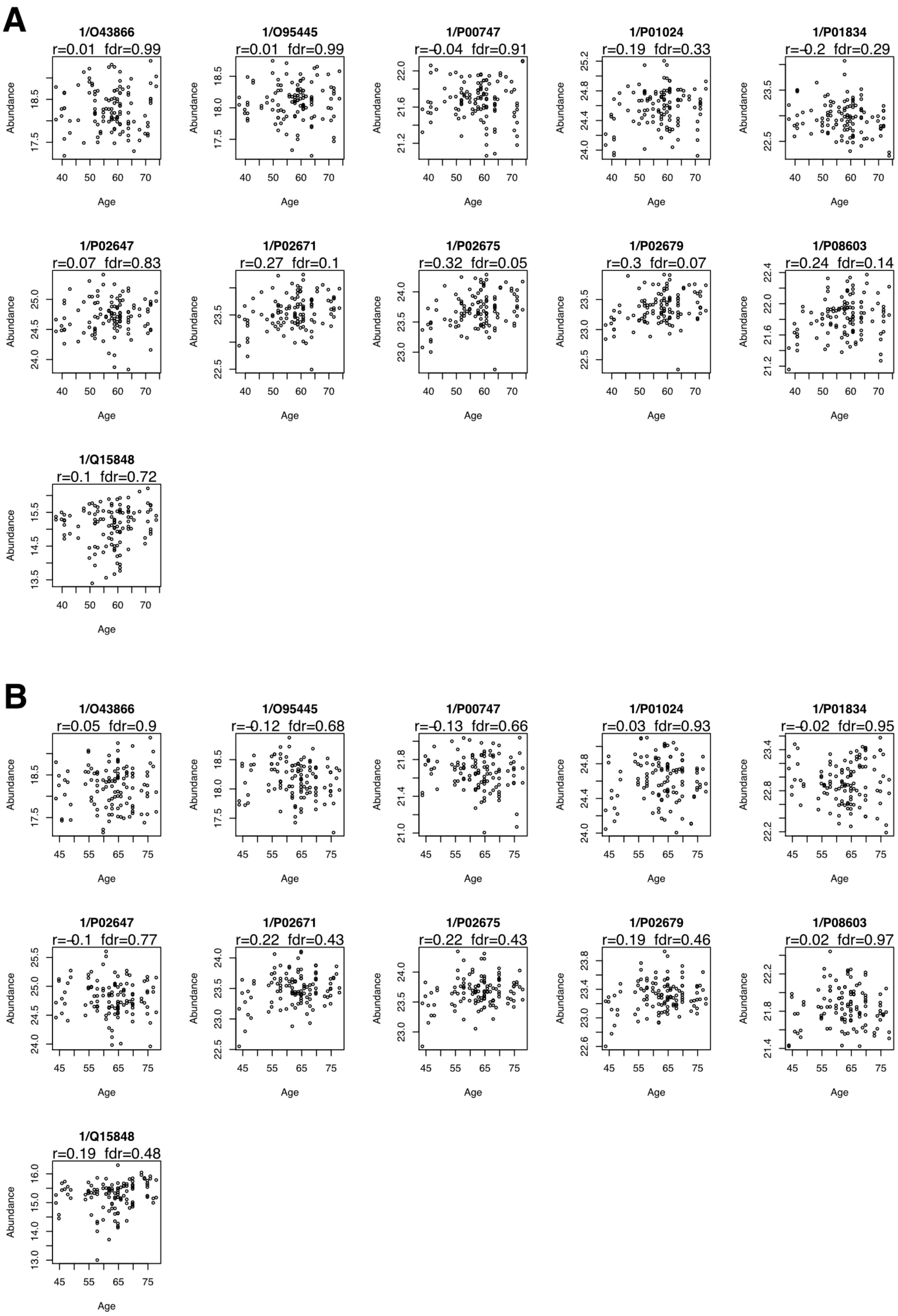

Supplementary Figure 5: Pearson correlation coefficients (r) between protein abundance and subject age and their Benjamini-Hochberg adjusted significances (FDR) at the first visit (A) and the second visit (B) for the differentially expressed proteins in T2D.

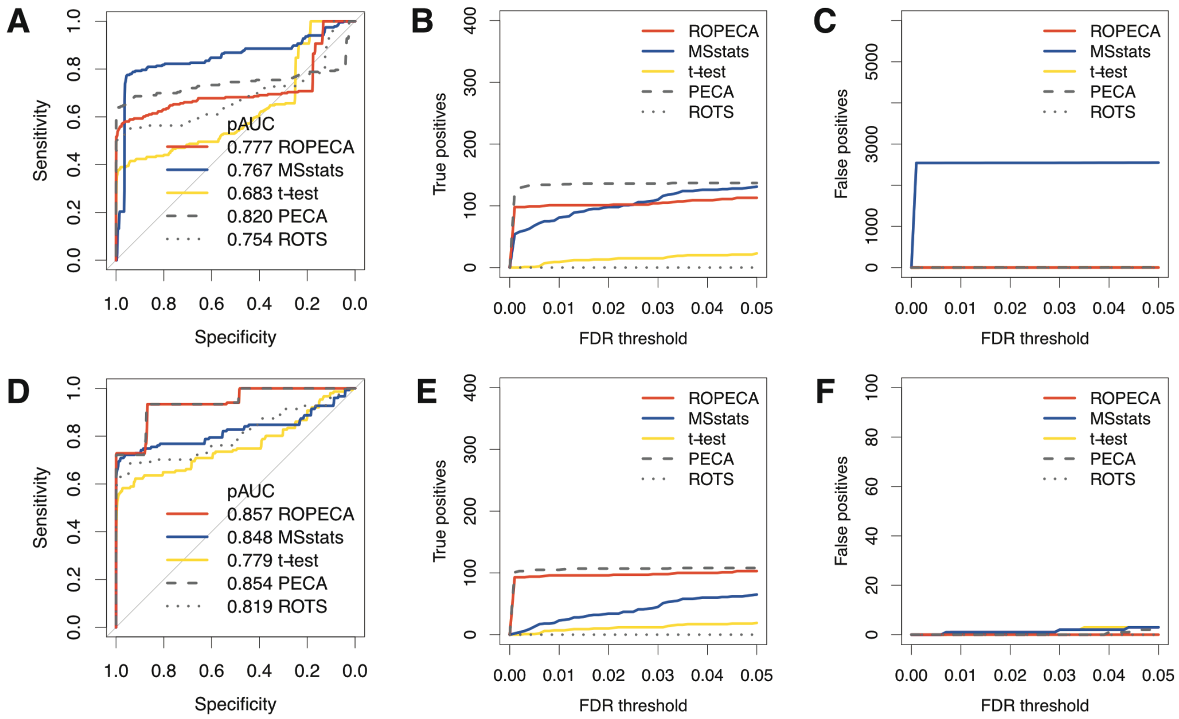

Supplementary Figure 6: Performance of ROPECA in the shotgun profiling standard data. **A**: Receiver operating characteristic (ROC) curves of the different statistical methods in the shotgun profiling standard data. The ROC curves were produced by merging the individual results of all pairwise comparisons. The partial area under the curve (pAUC) for specificity above 0.9 is shown for each method. **B, C**: Number of true positives (B) and false positives (C) in the shotgun profiling standard data as a function of FDR threshold. The large number of false positives reported by MSstats is due to proteins with missing values in one of the compared groups so that MSstats reports FDR as 0 and *p*-values as missing (*NA*). **D-F**: The same comparisons as in Figures A-C, but excluding those proteins for which ROPECA or t-test were unable to determine FDR due to too many missing values.
